# Sleep disturbance, dyspnea, and anxiety following total joint arthroplasty: an observational study

**DOI:** 10.1186/s13018-022-03288-x

**Published:** 2022-08-19

**Authors:** Steven Z. George, Michael P. Bolognesi, Sean P. Ryan, Maggie E. Horn

**Affiliations:** 1 Department of Orthopaedic Surgery, Duke University, 200 Morris Street, Durham, NC 27001 USA; 2 Duke Clinical Research Institute, Duke University, Durham, NC USA; 3 Division of Adult Reconstruction, Duke University, Durham, NC USA; 4 Division of Physical Therapy, Duke University, Durham, NC USA

**Keywords:** Total joint arthroplasty, Patient-reported outcomes, Sleep disturbance, Anxiety, Dyspnea, Chronic pain grade

## Abstract

**Background:**

Patient-Reported Outcomes Measurement Information System (PROMIS) domains for sleep disturbance, anxiety, and dyspnea have been under-reported for total joint arthroplasty (TJA). The aims of this study were to report postoperative differences for these domains based on TJA location and chronic pain state. We also investigated whether these domains were associated with physical function and pain interference outcomes.

**Methods:**

This was a retrospective, observational study of patients who underwent hip, knee, or shoulder TJA (primary and revision surgeries) at a single academic tertiary referral center. A subset of these patients completed an email-based survey for chronic pain grade (Chronic Pain Grade Scale-Revised) and sleep disturbance, anxiety, dyspnea, physical function, and pain interference (PROMIS short forms). Pre-operative and operative data were extracted from the electronic health record. Data analysis investigated PROMIS domains for differences in TJA location and chronic pain grade. Hierarchical linear regression determined associations of these domains with physical function and pain interference.

**Results:**

A total of 2638 individuals provided informed consent and completed the email survey. In the ANOVA models for sleep disturbance, anxiety, and dyspnea, there was no location by chronic pain grade interaction (*p* > 0.05) and no difference based on TJA location (*p* > 0.05). There were differences for chronic pain grade (*p* < 0.01). The poorest postoperative outcome score for each domain was associated with high impact chronic pain. Furthermore, sleep disturbance and dyspnea had the strongest associations with physical function and pain interference (*p* < 0.01).

**Conclusions:**

Sleep disturbance, anxiety, and dyspnea did not vary based on TJA location, but were associated with postoperative chronic pain grade. Sleep disturbance and dyspnea were strongly associated with commonly reported outcomes of physical function and pain interference. These findings provide guidance for those interested in expanding TJA outcome assessment to include sleep disturbance, anxiety, and/or dyspnea.

**Supplementary Information:**

The online version contains supplementary material available at 10.1186/s13018-022-03288-x.

## Introduction

Total joint arthroplasty (TJA), including knee, hip, and shoulder replacement, are among the most commonly performed orthopedic surgeries [1, 2]. Assessing postoperative outcomes is often determined using metrics such as rates of readmission, medical or surgical complications, and revision rates [3]. Additionally, patient-reported outcome measures (PROM) have become more common and are gradually being incorporated into hospital-based and payer requirements [4]. One reason PROM are being used more frequently as a primary outcome in orthopedic surgery is that they incorporate the patient’s perspective on health quality of life for clinically relevant domains like physical function, pain interference, and depressive symptoms [5–7]. One measurement platform, the Patient-Reported Outcome Measurement Information System (PROMIS), has been rapidly gaining traction in orthopedic outcomes [6, 8, 9]. PROMIS was developed with robust psychometric and validation methods using item-response theory [10–12]. Unlike many measures used in orthopedic surgery, PROMIS was not developed for a specific disease state, condition, or body region [13]. Accordingly, PROMIS has the advantage of being able to compare outcomes across different patient populations, allowing for the evaluation of outcomes across various orthopedic procedures and anatomic locations [13].

Indeed, a recent systematic review from Horn et al. [8] documented an increased use of PROMIS measures in orthopedic surgery. Eighty-eight studies were included in this review, with a notable increase in PROMIS reporting in orthopedic surgery occurring from 2013 (1 study) to 2018 (50 studies) [8]. The 3 most commonly reported PROMIS domains were physical function (71% of studies in review), pain interference (61% of studies in review), and depression (32% of studies in review) [8]. Importantly, there are other PROMIS domains relevant for assessing postoperative outcomes following TJA. For example, sleep disturbance (4% of studies in the review), anxiety (13% of studies in the review), and dyspnea (0% of studies in the review) all affect the quality of life of patients [8]. However, these PROMIS domains are underreported for many orthopedic patient populations. Further investigation of domains other than physical function and pain interference is especially warranted in high volume procedures like TJA to better understand the overall impact on patient quality of life [8, 14, 15].

Therefore, the purpose of this paper was to investigate the PROMIS domains of sleep disturbance, anxiety, and dyspnea following total hip, knee, or shoulder arthroplasty. First, we determined whether there were postoperative differences in these domains based on TJA location and postoperative chronic pain grade. Chronic pain grade was included in the analysis because of the importance of pain on an individual’s quality of life and determination of a successful surgical outcome [16–19]. Our hypothesis was that there would be no differences in PROMIS domains based on TJA location, but we expected higher anxiety, sleep disturbances, and dyspnea based on increased chronic pain grade. Secondly, we used multivariate analyses to determine how these domains of interest (sleep disturbance, anxiety, and dyspnea) were associated with postoperative PROMIS physical function and pain interference scores, which are more commonly reported in the literature as outcomes following orthopedic procedures [8]. We had no specific hypotheses regarding how these domains would be associated with physical function and pain interference. Information from these analyses could be used to inform decisions on whether it is warranted to include sleep disturbance, anxiety, and dyspnea as part of routine outcome assessment following TJA [7, 14].

## Patients and methods

### Study overview

This was a retrospective, observational study of patients who underwent a hip, knee, or shoulder TJA or TJA-related procedure (e.g., revision surgery) at any affiliated hospital or ambulatory surgery clinic in a single academic tertiary referral center. A subset of these patients completed an email-based survey regarding their current chronic pain state. This study was approved by the Duke University Institutional Review Board (Pro00104774), and this paper was reported following the STROBE guidelines [20].

### Participants

**Fig. 1:**
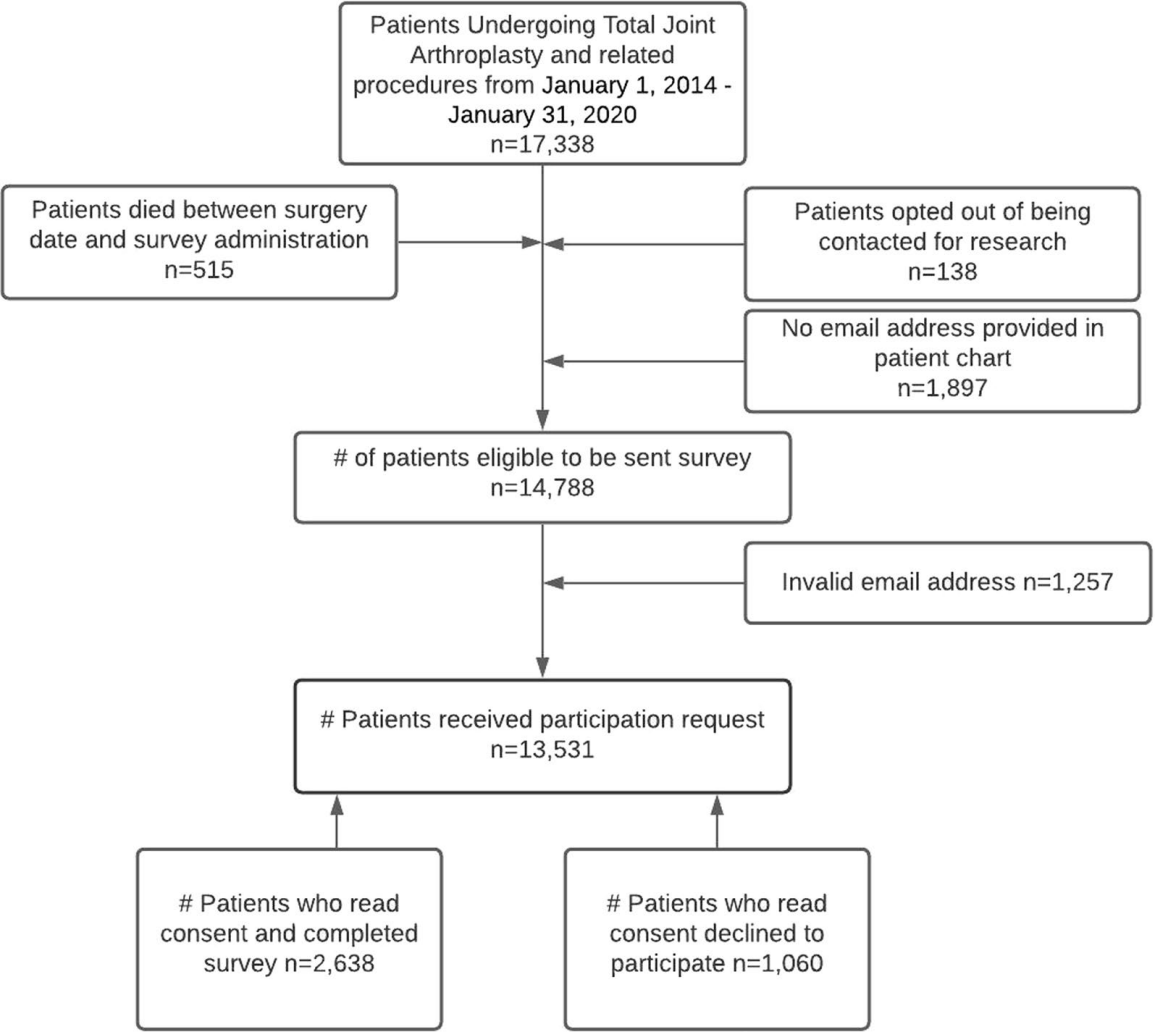
Flow for survey contact and completion

Eligible patients were identified through the electronic health record (EHR) (Epic Systems, Verona, WI) using a starting date of January 1, 2014 (i.e., the time in which there was widespread use of the EHR for this patient population) and an end date of January 31, 2020. The end date was selected to allow for at least 6-months from postoperative to first survey time (i.e., the minimum period for development of chronic pain). We identified 17,338 patients who underwent a TJA during this time period using current procedural terminology (CPT) codes (Additional file 1: Table S1). Patients were excluded if they died prior to January 31, 2020 (*n* = 515), opted out of being contacted for research (*n* = 138), did not have an email address on file (*n* = 1897), or had an invalid email address (*n* = 1257). After exclusions, a total of 13,531 patients were eligible to participate in the study (Fig. 1).

### Survey

An email survey was designed to collect self-reported information on chronic pain state, pain interference, and self-reported pain medication use. The eligible cohort was divided into 7 survey groups based on surgical year. Survey groups were created to allow for controlled distribution of the survey, which facilitated monitoring of response rates and the opportunity to respond to questions from potential participants in a timely manner. If patients underwent more than 1 surgery, the first surgery date was used for survey group assignment. For each survey group, a random number generator was used to determine the survey contact date. Random assignment for survey administration was used to avoid ordering effects that could confound survey responses (e.g., having higher response rates from those with more recent TJA). Patients were then sorted in ascending order by participant ID within each randomly determined group. The survey was administered in weekly waves beginning July 6, 2020, and ending on November 6, 2020.

Participants were contacted via email to provide informed consent to participate in the survey. The email indicated that the reason for this survey was to assess joint pain after receiving a TJA from the investigating institution. If patients did not complete the questionnaire on the first contact, 2 email reminders were sent 3 days apart. In order to increase response rates, the research coordinator contacted patients who consented to participate in the survey but had not initiated or completed the survey. In these cases, the research coordinator made a phone call to encourage survey completion. All survey information was collected by a link provided in the email that provided access to the secured survey.

### Measures

Information extracted from the EHR prior to surgery included age, sex, self-identified race, body mass index (BMI), tobacco use history, and comorbidity count. We also extracted surgical encounter information related to the TJA including preoperative pain rating (0–10 scale) within 30 days prior to surgery, the date of surgery, and the number of TJA or TJA-related surgeries performed within the data extraction period. These variables were selected based on having clinical relevance (e.g., preoperative pain intensity, number of surgeries) and/or prior association with TJA outcomes (e.g., BMI, age, tobacco use, comorbidity count, and sex). The length of time from first surgery to survey completion was selected to account for the variability in postoperative time.

Information from the survey responses included PROMIS short forms for physical function, pain interference, sleep disturbance, anxiety, and dyspnea. PROMIS measures have been validated for use in the general population [11, 12, 21, 22] as well as orthopedic surgery populations [14, 23–25]. Each PROMIS measure is standardized, such that a T score for each patient is given that has a mean score of 50 in the general US population and a standard deviation of 10 which enables comparison for clinical and research applications.

Information collected from the survey responses also included chronic pain status from the Graded Chronic Pain Scale-Revised which has been previously described in detail [26, 27]. Briefly, we used the Graded Chronic Pain Scale-Revised, which has 2 questions: (1) In the past 3 months, how often did you have pain? and (2) Over the past 3 months, how often did pain limit your life or work activities? These responses were combined with PROMIS pain interference score [21] to categorize patients into 4 pain states: (1) chronic pain absent (Question 1 response: “never,” or “some” days in pain); (2) mild chronic pain (Question 1 response: “most” or “every” day(s) in pain; Question 2 response: “never” or “some” days with impact on daily activities; Pain Interference Score < 50th percentile); (3) bothersome chronic pain (Question 1 response: “most” or “every” day(s) in pain; Question 2 response: “never” or “some” days with impact on daily activities; Pain Interference Score ≥ 50th percentile); and (4) high impact chronic pain (Question 1 response: “most” or “every” day(s) in pain; Question 2 response: “most” or “every” day(s) with impact on daily activities). This scale has been used recently to categorize rates of chronic pain in different populations, including the general population [28], those enrolled in a health care plan [26], those with spinal pain [29], and following TJA [30].

### Data management

Survey data were collected and managed using Research Electronic Data Capture (REDCap) electronic data capture tools hosted at the investigating institution. REDCap is a secure, web-based software platform designed to support data capture for research studies and provided an intuitive interface for validated data capture; audit trails for tracking data manipulation and export procedures; automated export procedures for seamless data downloads to common statistical packages; and procedures for data integration and interoperability with external sources [31, 32].

### Data analysis

Data analyses were completed with SPSS (IBM SPSS Statistics for Windows, Version 25.1, IBM Corp., Armonk, NY). Frequencies for those who responded to the email survey were compared who those who did not respond using chi-square analysis. Descriptive statistics were generated for the cohort who completed the email survey using mean (SD) for continuous variables and frequency (%) for categorical variables. Type I error rate was set at 0.05.

First, PROMIS sleep disturbance, anxiety, and dyspnea domains scores were compared by TJA location (hip, knee, and shoulder) and postoperative chronic pain status (chronic pain absent, mild chronic pain, bothersome chronic pain, high impact chronic pain) via ANOVA. Interaction (TJA location by chronic pain status) and main (TJA location or chronic pain status) effects were considered in the ANOVA models for each of the PROMIS domains.

Second, separate hierarchical linear regression models investigated the association of sleep disturbance, anxiety, and dyspnea with physical function and pain interference. Preoperative and surgical encounter data extracted from the EHR were included as continuous (age, preoperative pain intensity, BMI, comorbidity, and days from surgery to survey completion) or dichotomous variables (sex, race, history of tobacco use, TJA location, and number of surgeries). In the first block of the hierarchical regression models, the preoperative and surgical encounter data were all entered as control variables. In the second block of the hierarchical regression models, the PROMIS domains of interest (sleep disturbance, anxiety, and dyspnea) were all entered to assess their associations with physical function or pain interference.

Finally, we performed the same regression modeling as previously described but separately by TJA location. This was done as an exploratory analysis to determine if there were any PROMIS domains that had differential associations with physical function or pain interference based on TJA location.

## Results

During the study period, 2638 individuals provided informed consent and completed the email survey (Fig. 1). Survey participants did not differ based on age when compared with all individuals receiving TJA during the study period (64.4 [SD = 9.6] vs. 64.7 [SD = 1.1] years; p = 0.17). However, there were differences in survey participants for other variables. Male participants were more likely to respond than female participants (16.6% vs. 14.1%), individuals identifying as Caucasian/White were more likely to respond than those identifying as Non-White (17.9% vs. 5.6%), participants with hip and knee surgery were more likely to respond than participants with shoulder surgery (15.9% hip and 15.2% knee vs. 12.1% shoulder), and participants with 2 or more TJA-related surgeries were more likely to respond than participants with 1 surgery (17.1% 2 + surgeries vs. 14.7% 1 surgery) (all *p* < 0.001). Table 1 includes the descriptive statistics for the cohort included in the subsequent data analyses (*n* = 2638).

**Table 1:** Descriptive statistics for the TJA cohort

Variable	TJA cohort (*N* = 2638)
Data from medical record	
Age, mean (SD), yrs	64.4 (9.6)
Sex, no. (%)	
Female	1404 (53.3%)
Male	1234 (46.8%)
Race, no. (%)	
White	2432 (92.2%)
Non-White	206 (7.8%)
Tobacco use, no. (%)	
Never	1299 (49.2%)
Past or current	1017 (38.6%)
Pre-operative pain intensity, 0–10 scale, mean (SD)	3.6 (2.9)
BMI, mean (SD), kg/m^2^	29.9 (5.7)
Comorbidity count, mean (SD)	0.6 (0.9)
TJA location, no. (%)	
Hip	1146 (43.4%)
Knee	1265 (48.0%)
Shoulder	227 (8.6%)
Number of surgeries, no. (%)	
1	2037 (77.2%)
≥ 2	601 (22.8%)
Data from survey responses	
Survey time, mean (SD), days	176 (90.3)
Graded Chronic Pain Scale-Revised, no. (%)	
No chronic pain	1175 (44.5%)
Mild chronic pain	485 (18.4%)
Moderate chronic pain	693 (26.3%)
High impact chronic pain	285 (10.8%)
Sleep, mean (SD)	46.6 (8.9)
Anxiety, mean (SD)	47.6 (8.0)
Dyspnea, mean (SD)	30.3 (8.7)
Physical function, mean (SD)	47.9 (8.6)
Pain interference, mean (SD)	48.7 (8.9)

BMI, body mass index; SD, standard deviation; TJA, total joint arthroplasty

PROMIS measures for sleep, anxiety, dyspnea, physical function, and pain interference are scored on a standardized scale ranging from 0 to 100

### Postoperative differences by TJA and chronic pain grade

In the ANOVA models for sleep disturbance, anxiety, and dyspnea, there was no location by chronic pain grade interaction (*p* > 0.05) and no differences based only on TJA location (*p* > 0.05). However, there were differences based on chronic pain grade (Table 2). For each TJA location, the poorest postoperative outcome score for each domain was associated with high impact chronic pain. Better outcomes scores for sleep disturbance, anxiety, and dyspnea were associated with lessening chronic pain grades (Table 2).

**Table 2:** Sleep, anxiety, and dyspnea by TJA location and chronic pain grade

PROMIS domain	Sleep	Anxiety	Dyspnea
Hip arthroplasty (*n* = 1124)			
No chronic pain	42.9 (7.7)	45.8 (7.4)	28.0 (7.1)
Mild chronic pain	45.2 (7.4)	46.4 (6.9)	27.4 (5.5)
Bothersome chronic pain	49.7 (8.0)	49.9 (7.9)	31.5 (8.1)
High impact chronic pain	56.8 (9.0)	53.6 (9.5)	38.6 (13.6)
*p* value (by chronic pain grade)	< 0.001	< 0.001	< 0.001
Knee arthroplasty (*n* = 1224)			
No chronic pain	43.3 (7.8)	45.9 (7.2)	29.0 (7.9)
Mild chronic pain	44.9 (7.9)	45.8 (6.9)	27.9 (5.8)
Bothersome chronic pain	49.3 (7.8)	49.2 (7.7)	32.7 (8.9)
High impact chronic pain	55.4 (8.7)	54.2 (8.4)	35.9 (11.3)
*p* value (by chronic pain grade)	< 0.001	< 0.001	< 0.001
Shoulder arthroplasty (*n* = 223)			
No chronic pain	43.8 (8.4)	45.8 (7.7)	28.9 (7.7)
Mild chronic pain	46.8 (6.2)	44.8 (6.4)	27.1 (3.9)
Bothersome chronic pain	50.1 (8.6)	47.2 (7.8)	32.3 (8.8)
High impact chronic pain	55.4 (9.1)	53.3 (9.6)	42.7 (12.9)
*p* value (by chronic pain grade)	< 0.001	< 0.001	< 0.001

PROMIS measures were scored on a T-score metric, with standard population values for mean scores (50) and standard deviation (10). In this table a mean value above 50 indicates that for that chronic pain grade there would be higher sleep disturbance, anxiety, or dyspnea in that group compared to the general US population. A mean value below 50 would indicated lower values in comparison to the general US population

### Association with physical function

The full (first and second blocks) linear regression model for physical function explained 41% variance. The first block accounted for 12% variance and the second accounted for 29% variance (*p* < 0.01 for *R*^2^ change each block). Individual predictors in the full model for physical function are reported in Table 3 using standardized coefficients. Age, sex, preoperative pain intensity, and BMI had a statistical association with physical function (Table 3). Furthermore, sleep disturbance, anxiety, and dyspnea were each independently associated with physical function (Table 3). In the full model, sleep disturbance and dyspnea were the 2 variables with the strongest associations with physical function.

**Table 3:** Multivariate linear regression for physical function and pain interference

	Physical function	Pain interference
Total model	*R*^2^ = 0.41, *p* < 0.001	*R*^2^ = 0.36, *p* < 0.001
Medical record variables		
Age	** − 0.10**	− 0.01
Sex (0 = female)	**0.07**	0.03
Race (0 = white)	− 0.02	**0.06**
Tobacco use (0 = no history)	− 0.05	0.02
Pre-operative pain intensity	** − 0.03**	0.05
BMI	** − 0.10**	0.04
Comorbidity count	− 0.03	− 0.03
TJA location (0 = hip)	− 0.04	**0.07**
Number of surgeries (0 = one)	0.01	0.01
Survey response variables		
Survey time	− 0.03	0.02
Sleep	** − 0.31**	**0.35**
Anxiety	** − 0.11**	**0.17**
Dyspnea	** − 0.33**	**0.26**

Standardized coefficients are reported, bold font indicates *p* < 0.01

BMI, body mass index; TJA, total joint arthroplasty

### Association with pain interference

The full (first and second blocks) linear regression model for pain interference explained 36% variance. The first block accounted for 7% variance and the second accounted for 29% variance (*p* < 0.01 for *R*^2^ change each block). Individual predictors in the full model for pain interference are also reported in Table 3 using standardized coefficients. Race and TJA location had a statistical association with pain interference (Table 3). Furthermore, sleep disturbance, anxiety, and dyspnea were each independently associated with pain interference (Table 3). In the full model, sleep disturbance and dyspnea were the 2 variables with the strongest associations with pain interference.

### Regression models by TJA location (exploratory analysis)

Full model *R*^2^ values and standardized coefficients for sleep disturbance, anxiety, and dyspnea are reported separately for hip, knee, and shoulder arthroplasty in Table 4. These models show little evidence of differential associations between these PROMIS domains and physical function or pain interference. Sleep disturbance and dyspnea remained the strongest individual variables associated with physical function and pain interference for each location. One difference of note is that for shoulder arthroplasty dyspnea had a stronger association with the outcomes of interest than for hip and knee arthroplasty.

**Table 4:** Exploratory regression analysis by TJA location

	Physical function	Pain interference
Hip TJA		
Total model	*R*^2^ = 0.43, *p* < 0.001	*R*^2^ = 0.36, *p* < 0.001
Sleep	** − 0.32**	**0.37**
Anxiety	** − 0.09**	**0.12**
Dyspnea	** − 0.34**	**0.25**
Knee TJA		
Total model	*R*^2^ = 0.39, *p* < 0.001	*R*^2^ = 0.36, *p* < 0.001
Sleep	** − 0.31**	**0.34**
Anxiety	** − 0.12**	**0.19**
Dyspnea	** − 0.31**	**0.23**
Shoulder TJA		
Total model	*R*^2^ = 0.45, *p* < 0.001	*R*^2^ = 0.43, *p* < 0.001
Sleep	** − 0.26**	**0.26**
Anxiety	** − 0.13**	**0.12**
Dyspnea	** − 0.42**	**0.42**

Standardized coefficients are reported, bold font indicates *p* < 0.01

TJA, total joint arthroplasty

## Discussion

This paper reported on postoperative sleep disturbance, anxiety, and dyspnea following TJA for hip, knee, and shoulder using PROMIS measures. We investigated these domains because they are important for an individual’s quality of life, yet are not commonly used for outcome assessment in orthopedic surgery [16]. Our findings provide guidance for those interested in expanding outcomes assessment for TJA into domains beyond the more commonly reported domains of physical function and pain interference [7, 14].

One important element of outcome assessment is determining how a measure performs across different patient populations. Our first aim addresses this issue by comparing these PROMIS domains across 3 TJA locations and 4 different chronic pain states. Our findings indicated that similar postoperative levels of sleep disturbance, anxiety, and dyspnea were reported following hip, knee, or shoulder TJA. However, these PROMIS domains were strongly associated with the severity of chronic pain state. High impact chronic pain (the most severe pain state) and no chronic pain (the least severe pain state) were associated with the highest and lowest levels of sleep disturbance, anxiety, and dyspnea, respectively. Collectively these findings indicate that these PROMIS domains may be an appropriate outcome metric for analyses that include multiple procedure types. Based on the differences observed across chronic pain grades, these PROMIS domains would also be appropriate for those interested in factors contributing to postoperative chronic pain.

Another consideration for outcome assessment is to determine how the new measures relate to existing measures commonly used, or are currently considered clinical standards. Our second aim addressed this issue by investigating associations between sleep disturbance, anxiety, and dyspnea with physical function and pain interference. The findings from the multivariate linear regression models indicated that sleep disturbance and dyspnea were the domains with the strongest associations with physical function and pain interference. The finding for sleep disturbance converges with prior univariate analyses from the same academic tertiary care setting in which all patients seeking orthopedic care (i.e., not just for TJA) were analyzed [14]. The relatively weaker contribution of anxiety was somewhat surprising given our prior univariate analysis [14] and also the impact anxiety has on overall health for patients seeking orthopedic care [23]. These findings have 2 possible implications for expanding outcome assessment in TJA. First, those who are interested in learning more about variation in physical function and pain interference scores could consider adding sleep disturbance and dyspnea to existing outcome batteries. These measures could be considered as secondary outcomes and/or potential outcome mediators for physical function and pain interference. Second, those who are interested in adding outcomes that are not already associated with physical function and pain interference could consider adding anxiety to existing outcome batteries. Anxiety had relatively low associations with physical function and pain interference, so it would provide additional information on postoperative outcomes in comparison with sleep disturbance and dyspnea.

The strengths of this study include its sample size, surveying all patients within a single health system, inclusion of 3 TJA anatomic locations in the analysis, and collection of postoperative outcomes not commonly used in orthopedic surgery. The approach to administer the survey was robust in that an attempt was made to contact all eligible individuals that received a TJA during the study period. Even with this approach the overall response rate was low and differences in response rates were noted for sex, race, location of TJA, and number of surgeries. Most of these differences, while statistically detectable at 0.05 alpha level, were of small magnitude. For example, there was a 2.5% difference based on sex and a 3.8% difference based on TJA location. The difference based on race was notably larger—a 12.3% differential survey response for respondents that identified as white compared to those as identifying as non-white. Overall the implication of the survey response rates is that this sample was likely to be representative of the overall TJA population seeking care in this location, with the exception of race. For race it is likely that this sample has under representation of survey respondents that identify as non-white.

There are also several limitations to consider for this study. First, we did not include the PROMIS domain for depression in the survey due to concerns over patient burden. This domain would have been informative to the second aim by providing another measure related to mental health. We did not have any preoperative PROMIS scores for the domains reported in this analysis so we cannot comment on the change in outcomes that occurred with surgery. Second, there was high variability in the postoperative time completing the survey, ranging from 6 months to 5 years. This means that our findings cannot be generalized to a specific postoperative period (e.g., 1 year following TJA), although all follow-up periods were sufficiently long to determine chronic postoperative pain status. Third, is that the pre-operative data was collected from the electronic health record and did not include some variables that were likely associated with the outcomes of interest. For example; ethnicity, education completed, and physical activity levels were not routinely collected in this setting for those receiving TJA and could not be extracted for statistical analysis. A final limitation to consider is that patients completing the survey were contacted because they had a TJA but were not instructed to fill out the surveys only for TJA-related issues. Therefore, other body areas or limitations could be contributing to the overall scores for the PROMIS domains reported in this analysis.

The finding that the PROMIS dyspnea domain was strongly associated with physical function and pain interference was a novel finding that may merit confirmation in a separate cohort, and should be a consideration for future research. The systematic review investigating PROMIS measures in orthopedic surgery indicated no prior studies including dyspnea [8]. We know that cardiorespiratory complications remain a challenge in a small percentage of patients undergoing TJA but this has yet to be quantified with a standardized self-report measure like PROMIS. The current analysis included dyspnea and overall the levels of dyspnea were low in comparison with population means. Perhaps this is to be expected given that there has to be a certain level of cardiorespiratory health in order to be a candidate for TJA. However, even with the low levels of dyspnea observed in this cohort, dyspnea was associated with physical function and pain interference. Interestingly in the exploratory analyses, there was some evidence that for shoulder arthroplasty dyspnea may be a stronger contributor to physical function and pain interference than for hip and knee arthroplasty.

These findings should be considered as hypothesis generating and future studies are necessary to confirm these findings. In particular, the role of dyspnea in postoperative outcomes would be interesting to explore because this factor has high clinical relevance, yet is not frequently considered in studies of TJA. These findings also could be used to inform power analysis and sample size estimates for future prospective studies. In particular these data suggest that those interested in detecting post-operative differences in sleep, dyspnea, or anxiety based on TJA location are likely to need large samples because we observed small differences in PROMIS measures. These findings also suggest that power for detecting differences in these outcomes would be improved by knowing the chronic pain grade; which depending on the research question could be useful for planning a future study. Finally, our findings provide multivariate estimates of associations between sleep, dyspnea, and anxiety with physical function and pain interference. These estimates could be used to power future studies that use pre-operative variables to predict post-operative outcomes.

## Conclusion

In orthopedic surgery, the PROMIS domains of sleep disturbance, anxiety, and dyspnea have not been widely reported following TJA [8]. Our findings suggest that these outcomes are not expected to vary based on TJA location, but higher levels of sleep disturbance, anxiety, and dyspnea will be associated with more severe chronic pain states. These findings also indicated that postoperatively sleep disturbance and dyspnea were strongly associated with physical function and pain interference. These findings have notable importance given the challenges that exist related to postoperative cardiorespiratory complications after TJA. Collectively, these findings provide guidance for those interested in expanding TJA outcome assessment to include sleep disturbance, anxiety, and/or dyspnea [7, 14].

## Supplementary Information

**Additional file 1: Table S1**. CPT codes for identifying total joint arthroplasty procedures.

## Data Availability

The datasets used and/or analyzed during the current study are available from the corresponding author on reasonable request.

## Abbreviations

BMI: Body mass index
CPT: Current procedural terminology
EHR: Electronic health record
PROM: Patient-reported outcome measures
PROMIS: Patient-Reported Outcomes Measurement Information System
REDCap: Research Electronic Data Capture
TJA: Total joint arthroplasty
