# Supplementary material for: Sleep disturbance, dyspnea, and anxiety following total joint arthroplasty: an observational study
Source: J Orthop Surg Res. 2022 Aug 19;17:396. doi: 10.1186/s13018-022-03288-x (PMC9392244; doi:10.1186/s13018-022-03288-x)
Supplement: Supplementary file 1 — Additional file 1: Table S1. CPT codes for identifying total joint arthroplasty procedures. [file 13018_2022_3288_MOESM1_ESM.docx]

**Additional file 1: Table S1. CPT Codes for Identifying Total Joint Arthroplasty Procedures**

| **Hip Arthroplasty** |  |
| --- | --- |
| CPT Code | Description |
| 27120 | Acetabuloplasty; (eg, whitman, colonna, haygroves, or cup type) |
| 27125 | Hemiarthroplasty, hip, partial (eg, femoral stem prosthesis, bipolar arthroplasty) |
| 27130 | Arthroplasty, acetabular and proximal femoral prosthetic replacement (total hip arthroplasty), with or without autograft or allograft |
| 27132 | Conversion of previous hip surgery to total hip arthroplasty, with or without autograft or allograft |
| 27134 | Revision of total hip arthroplasty; both components, with or without autograft or allograft |
| 27137 | Revision of total hip arthroplasty; both components, with or without autograft or allograft |
| 27138 | Revision of total hip arthroplasty; femoral component only, with or without allograft |
| 27090 | Removal of hip prosthesis; (separate procedure) |
| 27091 | Removal of hip prosthesis; complicated, including total hip prosthesis, methylmethacrylate with or without insertion of spacer |
| **Knee Arthroplasty** |  |
| CPT Code | Description |
| 27440 | Arthroplasty, knee, tibial plateau |
| 27441 | Arthroplasty, knee, tibial plateau; with debridement and partial synovectomy |
| 27442 | Arthroplasty, femoral condyles or tibial plateau(s), knee |
| 27443 | Arthroplasty, femoral condyles or tibial plateau(s), knee; with debridement and partial synovectomy |
| 27445 | Arthroplasty, knee, hinge prosthesis (eg, Walldius type) |
| 27446 | Arthroplasty, knee, condyle and plateau; medial OR lateral compartment |
| 27447 | Arthroplasty, knee, condyle and plateau; medial AND lateral compartments with or without patella resurfacing (total knee arthroplasty) |
| 27486 | Revision of total knee arthroplasty, with or without allograft; 1 component |
| 27487 | Revision of total knee arthroplasty, with or without allograft; femoral and entire tibial component |
| 27488 | Removal of prosthesis, including total knee prosthesis, methylmethacrylate with or without insertion of spacer, knee |
| **Shoulder Arthroplasty** |  |
| CPT Code | Description |
| 23470 | Arthroplasty, glenohumeral joint; hemiarthroplasty |
| 23472 | Arthroplasty, glenohumeral joint; total shoulder (glenoid and proximal humeral replacement (eg, total shoulder)) |
| 23473 | 73 Revision of total shoulder arthroplasty, including allograft when performed; humeral or glenoid component |
| 23474 | Revision of total shoulder arthroplasty, including allograft when performed; humeral and glenoid component |
